# Communal breeding affects offspring behaviours associated with a competitive social environment

**DOI:** 10.1038/s41598-018-35089-w

**Published:** 2018-11-15

**Authors:** Stefan Fischer, Neus T. Pujol, Rhiannon Bolton, Jane L. Hurst, Paula Stockley

**Affiliations:** Mammalian Behaviour and Evolution Group, Institute of Integrative Biology, University of Liverpool, Leahurst Campus, Neston, CH64 7TE UK

## Abstract

Communal breeding is characterised by shared care of offspring produced by more than one female, and can affect the behavioural development of young. The decision to care communally can vary according to local conditions, and has been hypothesised to occur more frequently when social competition is intense. However, it is unknown whether communal rearing of young influences adult behaviours likely to be adaptive under competitive conditions. Here, using a controlled experimental approach, we investigate effects of communal rearing on competitive and exploratory behaviours of adult male house mice. In tests of competitive scent marking, only communally-reared subjects discriminated between related and unrelated rivals, depositing more scent marks in close proximity to unrelated males. Communally-reared subjects also displayed higher exploratory tendencies, with an increased probability of crossing a water barrier, while not exhibiting higher activity levels in an open field test. Since exploration tendencies and discrimination between kin and non-kin are likely to be advantageous when dispersing from the natal territory or in a high density population, our findings suggest that communal rearing prepares male house mice for a competitive social environment. Our results add to growing evidence that the early social environment influences development of important behavioural competences to cope with social challenges later in life.

## Introduction

Evidence of developmental plasticity, where organisms adapt to environments experienced during ontogeny, is widespread among animals^1–3^. Cues obtained through an individual’s own experience or via maternal effects can have life-long effects on adult behavioural phenotypes^4^. For example, high competition during early life, experienced by mothers or directly by offspring, might shape the competitive ability of offspring later in life, and potentially prepare them for a highly competitive environment. In support of this idea, it has been found that mothers experiencing intense social competition, in terms of breeding density or stability of social partners, produce more competitive offspring, often in a sex specific manner^5–8^.

In species with facultative communal rearing of young, early life experience will be strongly influenced by a mother’s decision either to nest communally or to rear her offspring alone. Communal rearing has been hypothesised to occur more frequently under conditions when social competition is intense^9–11^. However, it is not known if communally reared individuals develop a more competitive phenotype in preparation for more intense social competition. Studies investigating the influence of communal rearing on behavioural development in laboratory mice report that individuals reared in communal nests have a higher propensity to interact socially, are quicker to achieve a well-defined social role, and display more maternal care when rearing young themselves^12,13^. Pups in communal nests receive more care and engage in more peer-to-peer interactions^14,15^. Moreover, communal rearing is linked to an increase in oxytocin (OT) and arginine-vasopressin (AVP) receptors in certain brain areas. OT and AVP are nonapeptides mainly produced in the hypothalamus that mediate maternal, pair bonding, social and aggressive behaviours in many social mammals^16,17^. These differences are transmitted to the next generation without repeated experience of communal rearing conditions^13^. In addition to influencing the development of social behaviours, the early social environment also shapes anxiety-related behaviours, including exploration tendencies in rats and mice. For example, previous studies found that offspring receiving less maternal care display more anxiety-related behaviours^18–20^. Building on this work, it is important to consider how behavioural changes linked to communal rearing might function within the socially complex and competitive context of natural populations.

House mice (*Mus musculus domesticus*) live in flexible social units where multiple females within groups compete for access to resources and reproductive opportunities^9,21^. When competition is intense, females are more likely to raise their litters in communal nests where two or sometimes more females care for their pups, resulting in diverse fitness benefits for females^22–25^. While there are documented benefits of communal breeding, there is also evidence that it might be detrimental under certain circumstances, because of an increased risk of infanticide if litters are born asynchronously, or because a female’s investment into communally reared pups is dependent on the average litter size and not on her own litter size^26,27^. Females prefer to nest with closely related partners to rear young communally. Kin recognition is mediated by familiarity during rearing, by similarity of odours between related females, and by co-inheritance of species-specific specialized communication proteins in the urine (Major Urinary Proteins, MUPs) that indicates very close relatedness^28^. Thus, pups raised in communal or single nests might acquire important information about their current and future social environment, resulting in different developmental trajectories depending on their early social experience.

Male house mice often have greater dispersal tendencies than females, with young males more likely to leave the natal territory on reaching maturity^29^, (but see also^30^). When male offspring mature they start to receive elevated aggression from the dominant male, and may either stay in the natal territory as a subordinate waiting for an opportunity to breed, or disperse into a new area^31^. Dispersing males will be more likely to encounter and compete with non-relatives, whereas males remaining in the natal territory will more often be in competition with related males. Male house mice use scent marks to advertise their competitiveness and territory ownership^32^. Territory owners scent mark more than subordinates and counter mark the scent marks of territory intruders^31^. Thus, scent marking activity towards related and unrelated rivals, and the tendency to explore new territories, are both important behaviours regulated by male-male competition.

Here, we test for effects of communal rearing on the behaviour of mature male house mice. Using a controlled experimental approach, we reared subjects in communal or single nests to investigate how the rearing environment influences: (1) scent marking responses to related and unrelated rival males, (2) latency to cross a water barrier to reach a new territory, and (3) activity in an open field test. We predicted that the early social environment will: (1) shape individual competitiveness and (2) influence exploration tendencies that potentially underlie distinct life history trajectories.

## Results

### Social competition assay

**Figure 1:**
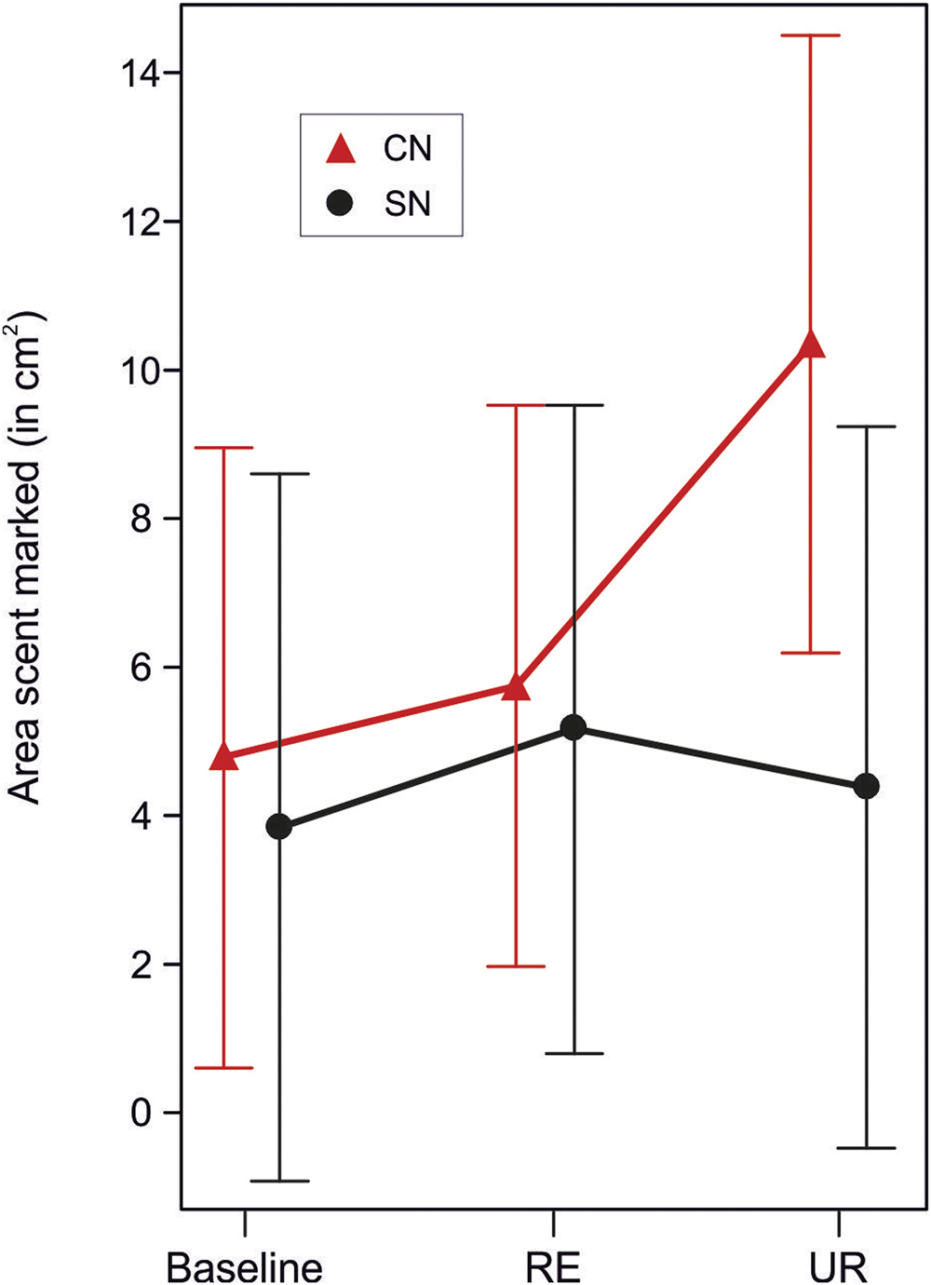
Total area scent marked (predicted values ± CI) closest to the stimulus male during the social competition assay. All test males scent marked a larger area when any (related, RE or unrelated, UR) stimulus male was present. Communal nest reared males (CN) scent marked a larger area in the presence of an unrelated stimulus male compared to single nest reared males (SN).

We first tested the competitive responses of subjects reared in communal (CN) or single (SN) nests by comparing the scent marks they deposited in response to related or unrelated rival stimulus males, or a control situation with no stimulus male present. For scent marks deposited close to the stimulus barrier, the response to these different stimuli varied according to the subject’s rearing background (interaction between stimulus and subject background: N = 68, F_2,46.2_ = 2.92, p = 0.06), justifying further exploration of these responses. Employing orthogonal contrasts, we found that this interaction between stimulus type and a subject’s rearing background is driven by an increase in scent marking activity of CN males in the presence of an unrelated opponent. Irrespective of their rearing environment, males marked a significantly larger area in the presence of any stimulus male compared to the control situation (Table 1a). Further, overall scent marking activity did not differ between CN and SN subjects when stimulus males were present (Table 1b) or absent (Table 1c). However, CN subjects marked a significantly larger area in the presence of an unrelated compared to a related stimulus male (Table 1d, Fig. 1), while the scent marking activity of SN subjects did not differ according to the relatedness of stimulus males (Table 1d, Fig. 1). Age of subjects did not influence the area covered by scent marks close to the opponent (N = 68, F_1,51.95_ = 1.14, p = 0.3) and was therefore not included in the orthogonal contrasts.

**Table 1:** Area covered by scent marks in the social competition assay when analysing the area closest to the barrier.

Factors	Estimate ± SE	Test statistic	*P*-value
Intercept	5.746 ± 1.362	4.218	**0.018**
**a. Stimulus male present versus absent**			
(CN-0, SN-0) vs. (CN-R, CN-U, SN-R, SN-U)	0.759 ± 0.359	2.115	**0.040**
**b. CN versus SN subjects (stimulus male present)**			
(CN-R, CN-U) vs. (SN-R, SN-U)	−1.538 ± 1.392	−1.105	0.335
**c. CN versus SN subjects (stimulus male absent)**			
CN-0 vs. SN-0	−0.508 ± 1.570	−0.324	0.757
**d. Related versus unrelated stimulus male (CN subjects)**			
CN-R vs. CN-U	2.306 ± 0.772	2.988	**0.004**
**e. Related versus unrelated stimulus male (SN subject)**			
SN-R vs. SN-U	−0.091 ± 0.819	−0.112	0.911

Orthogonal contrasts are presented which were performed after confirming that the interaction between treatment and background was significant (p < 0.1). Intercept estimate represents the grand mean of all treatments. Orthogonal comparisons of the treatments (a-e) are displayed as: CN-0 = communal nest reared – control; SN-0 = single nest reared – control; CN-R = communal nest reared – related opponent; CN-U = communal nest reared – unrelated opponent; SN-R = single nest reared – related opponent; SN-U = single nest reared – unrelated opponent. The direction of comparison within a contrast is left to right and the estimate value always refers to the treatment(s) to the right. If treatments are combined in parentheses, mean values of these treatments are used in the comparisons. N = 17 test males in 68 trials; P-values < 0.05 are highlighted in bold. Further details on the statistical analysis are provided in the Methods section.

The number of scent marks deposited close to the barrier did not differ according to the main effects of stimulus type (N = 68, F_2,46.38_ = 2.48, p = 0.1), rearing background (N = 68, F_1,3.17_ = 0.84, p = 0.42), and the interaction (N = 68, F_2,46.34_ = 0.76, p = 0.48). Overall, older subjects marked the area close to the barrier more frequently compared to younger subjects (N = 68, F_1,56.28_ = 5.51, p = 0.02).

Considering scent marks deposited across the whole arena, neither the main effects nor the interactions revealed a difference in the total number of scent marks deposited (treatment: N = 68, F_2,46.26_ = 1.48, p = 0.24; background: N = 68, F_1,3.65_ = 0.27, p = 0.64; interaction: N = 68, F_2,46.23_ = 1.23, p = 0.3) or area covered by scent marks (treatment: N = 68, F_2,46.19_ = 0.5, p = 0.61; background: N = 68, F_1,3.36_ = 1.17, p = 0.35; interaction: N = 68, F_2,46.17_ = 1.1, p = 0.34). However, irrespective of their rearing background, older subjects deposited more scent marks (N = 68, F_1,53.07_ = 8.65, p = 0.01) but did not cover a larger area (N = 68, F_1,51.28_ = 2.78, p = 0.1).

### Water-barrier assay

**Figure 2:**
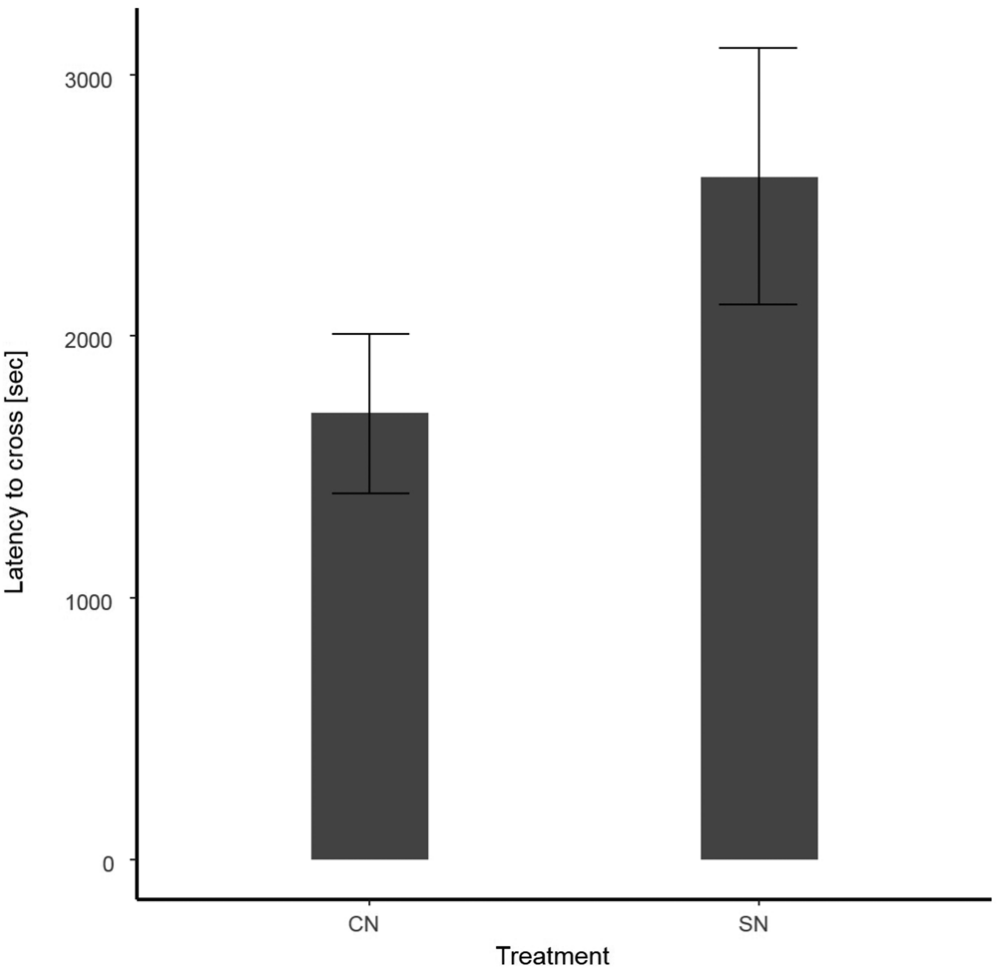
Latency to cross (mean ± SE) the water barrier in the water barrier assay. Communal nest reared males (CN) crossed the water barrier earlier compared to single nest reared males (SN).

Next we tested the willingness of subjects reared in communal versus single nests to cross a water barrier. Subjects reared in communal nests had a shorter latency to cross (Fig. 2, Table 2a), and were more likely to cross the water barrier (13 out of 14) compared to those reared in single nests (4 out of 10; N = 24, ${\chi }_{1}^{2}$ = 7.31, p = 0.007). Older subjects also crossed the water barrier later than younger subjects, irrespective of their rearing background (N = 24, ${\chi }_{1}^{2}$ = 5.17, p = 0.02, Table 2a).

**Table 2:** Parameters analysed in the water barrier and open field assay. Three parameters were recorded during the open field assay: (a) Latency to cross a water barrier in the water barrier assay.

Factors	Estimate ± SE	Test statistic	*P*-value
**a) Latency to cross a water barrier in the water barrier assay**			
Rearing background	−2.463 ± 0.911	7.31	**0.007**
Age	−0.266 ± 0.117	5.17	**0.02**
**b) Activity in the open field assay**			
Intercept	10.177 ± 0.274	—	—
Rearing background	0.166 ± 0.246	0.67	0.532
**c) Time spent in centre in the open field assay**			
Intercept	1.427 ± 0.107	—	—
Rearing background	−0.105 ± 0.107	0.832	0.372
**d) Latency to enter centre in the open field assay**			
Rearing background	−0.246 ± 0.432	0.32	0.57

(b) activity of subject males, (c) time spent in the centre, and (d) the latency to enter the centre of the open field arena. To obtain normal distributed residuals, we square-root transformed the activity and log-transformed the time spent in the centre of the open field arena. Estimates in (a) and (d) are based on treatment contrasts with CN as the reference and significance testing uses likelihood tests. Estimates for (b) and (c) are based on sum contrasts, and significance testing uses F statistics with a Kenward-Roger approximation for degrees of freedom. *P*-values < 0.05 are highlighted in bold.

### Open field test

Finally, we tested the behaviour of subjects reared in communal versus single nests in an open field test, to explore if differences in their willingness to cross a barrier might be linked to differences in general activity levels. None of the measured parameters in the open field test were significantly different between males experiencing a communal or single nest rearing environment. CN and SN males did not differ in their levels of activity (N = 24, F_1,19.41_ = 0.69, p > 0.1, Table 2b), time spent in the centre of the open field (N = 24, F_1,20.58_ = 0.46, p > 0.1, Table 2c) or their latency to enter the central area (N = 24, ${\chi }_{1}^{2}$ = 0.45, p > 0.1, Table 2d). Subject age did not influence any parameters analysed (all p values > 0.1) and was dropped from the final models.

## Discussion

Our results suggest that rearing male house mice in communal nests influences adult behaviours associated with a competitive social environment. At maturity, communally reared subjects were more competitive towards unrelated opponents, and more explorative in crossing a water barrier to reach a new territory. By contrast, early social experience did not influence overall scent marking rates or activity levels.

In support of our first prediction that the competitive behaviour of male house mice will be shaped by their early social environment, we found that subjects reared in communal nests scent marked a larger area in close proximity to an unrelated compared to a related rival. Since overall levels of scent marking and responses to related or unrelated males were not significantly different in single nest reared subjects, the elevated scent marking rate of communally reared males appears specifically directed to unrelated competitors. This behaviour could be explained if communally reared males are adapted for competing in high-density populations. For example, unrelated males may pose a particular threat of territory take-over under high density conditions^31^, and encounters with unrelated males are likely to be more frequent for individuals dispersing from the natal territory. Nonetheless, a degree of caution is required in interpreting these findings. Notably, to achieve a balanced experimental design required careful matching of subjects with suitable related and unrelated opponents, and this necessarily restricted our choice of subjects. Consequently, although the number of individuals tested provides reasonable power to detect an effect of treatment group on behaviour, the subjects used for this part of the study originated from a relatively small number of family groups, thus ultimately reducing their independence (see Table S1). Ideally therefore, further experimental investigation should be conducted to confirm the generality of this result across a more genetically diverse group of subjects. Notwithstanding this constraint however, it is also important to emphasise the vastly greater level of genetic diversity present in the randomly outbred wild-stock house mice used in this study compared to the laboratory mouse strains more typically used in previous similar investigations.

Although we did not attempt to quantify the physiological mechanisms underlying this behavioural response, communal rearing has previously been linked to changes in oxytocin and vasopressin receptor binding levels in several brain regions of female BALB/c laboratory mice^13^. Oxytocin and vasopressin are two neuropeptides known to influence social behaviour in many species^33^. In particular, oxytocin and vasopressin are important components in regulating mammalian social recognition during mother-infant and pair bonding^34^. The pivotal role of these peptides during social recognition is underlined by more recent evidence connecting the oxytocinergic system to in-group favouritism and out-group aggression in humans and chimpanzees^35,36^. Here, oxytocin has been linked to an interesting dual function of simultaneously increasing aggression and affiliation depending on the social context. Thus, if similar effects of communal rearing occur in male as well as female house mice, oxytocin could potentially be involved in mediating the elevated competitive response to unrelated opponents in our experiment. Alternatively, mice may acquire different abilities to differentiate between related and unrelated opponents according to their rearing background. In mice, individual and kin recognition is mediated by genetically determined scent signatures, which are strongly influenced by a set of polymorphic communication proteins termed major urinary proteins (MUPs)^28,37–39^. Communal reared males are likely to be exposed to a larger diversity of MUP and volatile signatures during rearing than those reared in single litters. Whether this leads to greater sensitivity in differentiating between related and unrelated opponents among communally reared mice thus warrants further investigation.

We also find support for our second prediction that exploration tendencies of male house mice are linked to early social experience. That is, communally reared males were more willing to cross a water barrier than single nest reared subjects, showing significantly shorter latencies to cross the barrier and reach a previously unexplored area. Water barriers have been used previously to test dispersal tendencies in mice^40–42^, and so an increased motivation to disperse among communally reared males is a possible interpretation of our findings. Under natural conditions, male house mice may choose to disperse from their natal group, mainly because of elevated aggression from the dominant territorial male or other siblings^29–31,40^. In our experiment there was no competitive pressure for subjects to disperse, since each was singly housed without any competitors. Hence the greater willingness of communally reared subjects to cross a water barrier may have been motivated by short-term exploration of a new environment rather than permanent dispersal to find an unoccupied territory to breed. Nonetheless, we found no evidence of differences in general activity levels as measured in the open field assay, in agreement with similar tests using BALB/c laboratory mice reared in communal or single nests^13^. The greater willingness of communally reared subjects to cross a water barrier in our study might therefore be interpreted as indicative of a more risk prone, dispersive phenotype. Alternatively, instead of measuring similar behavioural characteristics linked to anxiety and exploration, the open field and water-barrier assay could be testing different forms of anxiety behaviour. State anxiety is defined as the anxiety a subject experiences at a particular moment, and is tested by placing individuals in an unfamiliar environment, as in our open field test. By contrast, trait anxiety is considered as a permanent feature of an individual^43,44^ and is tested by giving animals the choice between a familiar and a novel area, as in our water barrier assay. Hence it is possible that in our experiment the early social environment might have shaped the permanent aspect (i.e. trait anxiety) but not the short-lived and situation-dependent aspect of anxiety (state anxiety) in subjects. This idea is supported by Kloke, *et al*.^44^ showing that communal rearing in laboratory mice influenced behaviours linked to trait anxiety but not state anxiety. Nevertheless, although the open-field test^45,46^ is commonly used to quantify exploratory and anxiety-like behaviours in laboratory rodents^47,48^, comparing behaviours between laboratory and wild mice can be problematic as they differ quantitatively and qualitatively in their strategies to assess risk^49^. Our results therefore underline that the water barrier test could be a more appropriate behavioural assay to investigate exploratory and anxiety behaviours in wild house mice.

The early social environment of pups raised in communal or single nests differs in at least three key respects, each of which might potentially have contributed to the different patterns of behaviour we report here: (1) mother-offspring interactions, (2) peer-to-peer interactions and (3) the level of competition among pups. For example, it is well established that pups raised in communal nests receive more maternal care and engage more in peer interactions^12^, and it is also likely that pups in communal nests will experience higher levels of competition as a result of larger litter sizes and the relative asynchrony in ages of the different litters^50,51^. Since our experiment is not designed to disentangle these different aspects of naturally formed communal versus single nest environments, further investigation will be required to determine the relative importance of each in explaining the behavioural differences we have reported. Based on natural variation in litter sizes, we could only look for evidence of relationships between litter size and the main results *within* each rearing background (i.e. separately within the CN and SN treatment groups respectively, see Figs S1–S4 in Supplementary Material). Although this allows us to speculate that differences in litter size *per se* might not be the main driver of the behavioural differences we report for communal and single reared males, in order to properly understand this will require a targeted experimental approach to disentangle the various potential effects of maternal and peer-related interactions.

Because our experiment maintained social contact between dams of single nest reared young, it is unlikely that differences in the behaviour of communal or single nest reared young result from social isolation of dams. Social isolation is stressful for female mice (see^52^ for a review) and maternal stress could influence behavioural development^53,54^. Hence in our experimental design, single rearing females were able to interact (through a mesh divider) with a familiar sibling, simulating a natural group with multiple breeding females sharing the same area. Our design thus differs from previous studies investigating the effects of communal rearing on behavioural and neural profiles of offspring, in which single rearing mothers have been isolated from other social partners^13,14^. The benefits of our design are twofold by: (1) increasing the welfare of females when breeding in a single nest; and (2) removing social isolation as a confounding variable, so that effects of the communal nest environment can be studied within an adaptive context.

In conclusion, we found significant influences of communal rearing on adult behavioural phenotypes in wild-derived male house mice. Subjects reared in communal nests were more competitive towards unrelated males and more explorative, without differences in general activity. Our study highlights that communal nesting might prepare offspring for a more competitive social environment and thus shapes important behavioural competences to deal with social conflict.

## Methods

### Subjects

Subject males (N = 41) were from a captive colony of house mice, derived from wild ancestors originating from several populations in the northwest of England, UK, with regular addition of new wild-caught animals. Most subjects used in the current study had ancestors bred with wild-caught animals within the previous one to three generations. The colony is maintained under controlled environmental conditions, with temperature 20–21 °C, relative humidity 45–65%, and a reversed 12: 12 h light cycle (lights off at 08:00). All animals are provided with *ad libitum* access to water and food (Lab Diet 5002 Certified Rodent Diet, Purina Mills, St Louis, MO, USA), and housed on Corn Cob Absorb 10/14 substrate with paper wool nest material. Subjects were bred in standard laboratory cages (MB1, North Kent Plastics, UK; 45 × 28 × 13 cm) with some modifications (detailed below). To obtain subjects reared in communal and single nests, we selected healthy and mature parental females (N = 16) and males (N = 8) from the breeding colony. These were assigned into eight breeding trios, each consisting of one full-sister pair and one unrelated male. With this breeding design we created a typical social structure for house mice with one dominant breeding male and several breeding females^9^. Sister pairs and their combined offspring at each breeding attempt (communal or single, see below) are hereafter referred to as family units. Thus, experimental animals raised in the same family unit were familiar full siblings (r = 0.5) or three-quarter siblings (i.e. same sire but dam is an aunt, r = 0.375). Experimental animals derived from different breeding trios did not share full-sibling grandparents (r < 0.032). Our experiment was designed to compare the behaviour of offspring produced by the same breeding trio under communal (CN) or single nest (SN) rearing conditions. Hence the same female pairs were allocated to both single and communal nest treatment groups in sequential breeding attempts, with balance for the order in which communal or single nest reared litters were produced. In one case, only one female gave birth in the communal nest treatment, and we classified the breeding attempt as a single nest treatment, even though this litter is likely to have experienced some differences in its early social environment compared to the other single nest reared litters. However, only one male offspring was used from this breeding trio, for the water barrier and open field assay, and we have checked that removing this subject from the analysis does not qualitatively change the results. As not all females bred successfully each time, breeding trios were bred up to 3 times, each female contributing a maximum of two litters to the same treatment group (see Table S1). Parental origin was taken into account in all analyses.

To reduce aggression and to stimulate females, all members of a breeding trio were primed with each other’s odours before being introduced. Each breeding trio was initially housed in a standard MB1 cage for one week. Female pairs were then randomly allocated to either SN or CN rearing treatments, and transferred to experimental MB1 cages to rear their litters until weaning. Experimental breeding cages were each identical in size and content, containing bedding, feeders, water bottles, and two nest boxes (13.7 × 9.3 × 7.2 cm). In SN breeding cages, the females were housed on either side of a mesh divider, separating the cage into two equal sized areas, each with a single nest box. The females were thus able to interact with each other, avoiding social isolation, but were prevented from forming a communal nest. In CN breeding cages, there was no divider and females could interact freely. The two nest boxes were combined to create one communal nest, with equivalent nest box space per female as in the SN cages. To create naturalistic conditions we did not interfere with the size or composition of the litters produced. Thus, when females were allowed to breed communally, litters were on average larger (CN = 11.1 ± 0.84; SN = 5.6 ± 0.5 [average number of pups ± SE]) and consisted of pups born on different days (CN = on average 2.2 days apart [min: 0, max: 5 days]). To improve welfare and to reduce the risk of abortion we did not disturb the lactating mothers until PND 14. By this stage it was not possible to assign the pups to their respective mothers according to their age or weight differences. Nevertheless, SN mothers were also kept together with a sister in the same cage (but separated by a mesh divider) and the spacing of birth within SN cages was comparable to the spacing of births in CN cages (SN = on average 2.2 days apart [min: 1, max: 3 days]). Our experimental design also assured that pups reared in CN and SN cages were kept under similar densities irrespective of litter size variations between the rearing treatments.

Experimental litters were produced in three blocks, with each sister pair assigned to a CN or SN cage, depending on their previous litter. In total, we obtained 32 litters from 15 females and a total of 179 pups. Eighteen litters containing 58 males and 42 female offspring from 14 dams were produced in communal nests and 14 litters containing 35 males and 44 female offspring from 12 dams were produced in single nests. All litters were weaned on post-natal days (PND) 28–30. Weaning weights of males were not different between communal and single nests (N = 92, t = −0.62, p = 0.54; CN = 13.3 ± 2.0; SN = 13.6 ± 2.0 [in g, average ± SD]). Male subjects were transferred individually into M3 cages (48 × 15 × 13 cm) until the end of the experiment.

### Behavioural assays

Behavioural assays were conducted after males reached sexual maturity to analyse (1) competitiveness, quantified as scent marking activity during a social competition assay, and (2) exploration tendencies, quantified as i) latency to cross a water barrier and ii) activity in an open field assay. Behavioural assays were conducted after subject males had reached sexual maturity (social competition assay: average age = 159, range = 104–472; water barrier assay: average age = 76.5, range: 64–90; open field assay: average age 161.2, range = 120–199 [in days]). For each assay we used two male offspring from each family unit, where available. For SN reared subjects we used one male offspring of each sister in the family unit. As we were not able to reliably distinguish the offspring of different sisters in communal nests (three-quarter siblings, r = 0.37), for CN reared subjects we randomly selected two males per family unit. All behavioural assays were recorded and observers were blind to the rearing background of subjects during the analysis. Further details of the behavioural assays are contained in the Supplementary Material, including a detailed overview of sample sizes in each behavioural assay (Table S1).

#### Social competition assay

Subject males (N = 17) were randomly selected from a total of 10 communal and seven single nest litters. We were constrained by the number of litters we could use because our experimental design matched subjects from litters reared in a given treatment with unfamiliar siblings reared in the opposite treatment. As a result, subjects for this component of the study each originated from four breeding trios providing 10 communal nest litters, and two breeding trios providing seven single nest litters (for exact sample sizes see Table S1). The assay was designed to investigate the competitiveness of males by recording their scent marking activity in response to encountering unfamiliar stimulus males that were (i) unrelated or (ii) related, as well as (iii) a control situation when no stimulus male was present. Unrelated (grandparents were not full siblings) and related (unfamiliar full siblings or three-quarter siblings) stimulus males were derived from different or the same breeding trios, respectively. Related and unrelated stimulus males were always age matched (average age for related: 205.4 and for unrelated: 205.4 days) and were either older (30 out of 51 trials with an opponent present) or younger than subjects (CN: older = 16, younger = 12; SN: older = 14, younger = 9). Each male received all three treatments (control, unrelated stimulus male, related stimulus male) in a randomly assigned sequence over a period of up to 5 days, with one trial (20 min) per day for each male. Scent marking activity was quantified as the area covered by scent marks and the number of scent marks deposited, both over the entire arena (25 × 41 cm) and in the quartile of the arena closest to the opponent (6.2 × 41 cm).

#### Water-barrier assay

Subject males (N = 24) were randomly selected from 10 SN litters and 16 CN litters and tested for exploratory behaviours when encountering a water barrier to reach an unknown area. Subjects were derived from all eight breeding trios (see Table S1). For this assay two standard laboratory cages were connected with a plastic water bath via transparent Perspex tunnels so that subject males could only enter the new cage by passing through a water bath. Directly after the water bath was connected to the second cage the observer left the room and the location of each subject was recorded for 1 h. Recordings were then used to analyse the latency of each subject male to reach the other side of the water barrier.

#### Open field assay

The same subject males (N = 24) as in the water barrier assay were transferred to an open field arena and released close to a side wall. The observer immediately left the room and the movement of each subject was recorded for 5 min. The recordings were used to analyse the (1) activity, and (2) exploratory behaviours of each male.

#### Statistical analysis

For statistical analysis, we used R 3.4.0^55^ with the packages ‘lme4’^56^, ‘afex’^57^ and ‘survival’^58^. We used linear mixed effect models (LMMs) and Cox-proportional-hazard regression models (COXPH). In all models, we used a family unit ID as a random effect for animals reared by the same breeding trio. If males were repeatedly tested (i.e. social competition assay) we also included animal ID as a random factor. We always fitted the rearing background (CN or SN) as a fixed effect with CN set as the intercept. In the social competition assay we also included the main effect of treatment (control (0), related opponent (R), and unrelated opponent (U)) with the control set as the intercept as well as the interaction between background and treatment as fixed factors. To minimize Type I error when analysing the social competition assay we used orthogonal contrasts (see^59^) if the p value of the interaction between the treatments and background was p < 0.1. First, we set the contrast of the model to compare the mean of both control situations with the mean of all treatments in which any opponent was present [(CN-0, SN-0) vs. (CN-R, CN-U, SN-R, SN-U)]. Second, we compared if the two control situations were different [CN-0 vs. SN-0]. Third, we compared the scent marking activity between communal and single reared males when any opponent was present [(CN-R, CN-U) vs. (SN-R, SN-U)]. Fourth, we compared whether communal reared males differed in their scent marking activity in the presence of a related or unrelated stimulus male [CN-R vs. CN-U]. Finally, we compared whether single reared males differed in their scent marking activity in the presence of a related or unrelated stimulus male [SN-R vs. SN-U]. Note that mean values of treatments presented in round brackets were used in the comparisons. Age of subjects was always included as a covariate but, if not significant, it was removed from the final models. Litter size and rearing background were statistically correlated because communal nests were always composed of larger litters (CN = 11.1 ± 0.84; SN = 5.6 ± 0.5 [mean ± SE]; t = 5.6; p < 0.001).

Residuals and Q/Q-plots of all LMM models were visually inspected and the distributions of residuals were compared to a normal distribution using Kolmogorov-Smirnov and Shapiro tests. If residuals were non-normally distributed a square-root (for number of scent marks in the social competition assay and the activity in the open field assay) or a log transformation (for time spent in centre of the open field) was applied and residuals again checked. In COXPH models, we validated the proportional hazards assumption for a Cox regression model fit. To obtain p-values of LMMs model fixed effects, we used the mixed() function in the package ‘afex’ with a Kenward-Roger approximation for degrees of freedom.

#### Ethical note

All procedures involved in this study were non-invasive behavioural tests. Animal use and care was in accordance with the EU directive 2010/63/EU and UK Home Office code of practice for the housing and care of animals bred, supplied or used for scientific purposes. The University of Liverpool Animal Welfare Committee approved the work, but no specific licenses were required. More details are provided in the Supplementary Material.

## Electronic supplementary material

Electronic Supplementary Material

## Data Availability

The data and R-codes are deposited to FigShare, available at https://dx.doi.org/10.6084/m9.figshare.6580349.
