# Supplementary material for: Communal breeding affects offspring behaviours associated with a competitive social environment
Source: Sci Rep. 2018 Nov 15;8:16850. doi: 10.1038/s41598-018-35089-w (PMC6237865; doi:10.1038/s41598-018-35089-w)
Supplement: Supplementary file 1 — Electronic Supplementary Material [file 41598_2018_35089_MOESM1_ESM.pdf]

Electronic Supplementary Material for: Communal breeding affects offspring behaviours associated with a competitive social environment

Stefan Fischer, Neus T. Pujol, Rhiannon Bolton, Jane Hurst & Paula Stockley

Electronic Supplementary Material include supplementary details on material and methods and supplementary results

Supplementary Material and Methods

Behavioural assays

Behavioural assays were conducted to analyse (1) competitiveness, quantified as scent marking activity during a social competition assay, and (2) exploration tendencies, quantified as i) latency to cross a water barrier and ii) activity in an open field assay. For each assay we used two male offspring from each family unit, if available. For single nest (SN) reared subjects we used one male offspring of each sister in the family unit. As we were not able to reliably distinguish the offspring of different sisters in communal nests (three-quarter siblings, r = 0.37), for communal nest (CN) reared subjects we randomly selected two males per family unit. For a detailed overview of sample sizes in each behavioural assay please refer to Table S1. Behavioural assays were conducted after subject males had reached sexual maturity (social competition assay: average age = 159, range = 104 – 472; water barrier assay: average age = 76.5, range: 64 - 90; open field assay: average age 161.2, range = 120 – 199 [in days]). Before each test subjects were weighed and, except for the open field test, stimulated with female scent the day before. All assays were conducted under red light between 0900 and 1700 h, in the same room used for housing the animals. Each trial was recorded with an overhead camera (Panasonic WV-BP330), connected to a DVD recorder (Panasonic DMR-E85H) and a monitor in a different room. Equipment was cleaned thoroughly after each use with hot soapy water and 70% ethanol. The observer was always blind to the rearing background of the test animal when analysing the recordings.

Social competition assay

Subject males (n= 17) were randomly selected from 10 communal and seven single nest litters. We were constrained by the number of litters we could use because our experimental design matched subjects from litters reared in a given treatment with unfamiliar siblings reared in the opposite treatment. As a result, subjects for this component of the study each originated from four breeding trios providing 10 communal litters and two breeding trios providing seven single litters (for exact sample sizes please refer to Table S1). The assay was designed to investigate the competitiveness of males by recording their scent marking activity in response to encountering unfamiliar stimulus males that were (i) unrelated or (ii) related, as well as (iii) a control situation when no stimulus male was present. Unrelated (grandparents were not full siblings) and related (unfamiliar full siblings or three-quarter siblings) stimulus males were derived from different or the same breeding trios, respectively. Thus, most subject males were tested with a stimulus male raised in the opposite treatment (15 out of 17). The rearing background of the stimulus male did not influence the scent marking activity of subject males ($N = 17$, $F_{1,32.4} = 1.29$, $p = 0.265$). Related and unrelated stimulus males were always age matched (average age for related: 7.34 and for unrelated: 7.33 month) and were either older (30 out of 68 trials) or younger than test males. Each male received all three treatments (control, unrelated stimulus male, related stimulus male) in a randomly assigned sequence over a period of up to 5 days with one trial per day for each male. The assay was conducted in an MB1 cage divided into two equal sized halves by a mesh barrier, which allowed visual and olfactory contact while preventing direct physical interaction. Each side of the cage was lined with Benchkote to record the scent marking activity of the test males. Each trial lasted 20 minutes and was monitored remotely in a separate room (see Ethical note below). Immediately after each trial the scent marked Benchkote was photographed in a dark box (40 x 56.5 x 37.5 cm) under UV light emitted by two strip lights. Subsequently, the number and area covered by scent marks was

quantified using the free software ImageJ (https://imagej.nih.gov/ij/), focusing on (1) the entire area of the cage (measurement 25 x 41 cm), and (2) the area closest to the opponent. To obtain the area closest to the opponent we divided each Benchkote in four equal sized zones (6.2 x 41 cm)

Water-barrier assay

Subject males (n=24) were randomly selected from 10 SN litters and 16 CN litters. The assay was conducted using modified MB1 cages with a 5 cm hole on the long side, containing substrate, nest material and *ad libitum* access to food and water. The hole was sealed using a rubber plug during an initial 48 h of habituation phase for each subject male. At the start of each test, assay cages were connected to a plastic water bath (54 x 14.5 x 1.4 cm, filled with 6 cm deep water and a Perspex lid on top) via a transparent Perspex tunnel. To reduce the gap between the water and the end of the tunnel a small wire mesh ramp was provided. This allowed subjects to explore and descend gradually into the water. The opposite end of the water bath was connected in the same way to a second identical MB1 cage, also providing substrate, nest material and *ad-libitum* food and water. Thus, the only way the males could enter the new cage was by passing through the water bath. Directly after the water bath was connected to the second cage the observer left the room and the location of each subject was recorded for 1 h. Recordings were then used to analyse the latency of each subject male to reach the other side of the water barrier. Subject males that did not cross the water barrier within the observation period were assigned with the maximum latency of 3600s (7 out of 24 males).

Open field assay

The same subject males (n=24) were used as in the water barrier assay. The open field arena had a rectangular shape measuring 69.7 x 60 cm with 54.9 cm high walls surrounding it. To start the open field assay, a male was transferred to the experimental apparatus and released close to a wall. The

observer immediately left the room and the movement of each subject was recorded for 5 min. To analyse the recordings, the arena was divided into a grid of nine equal-sized squares (23.2 x 20 cm), with one central square surrounded by eight peripheral squares. For each 5 min trial, we recorded (1) activity, as the number of line crosses of the grid, and (2) exploratory behaviour, as i) the latency to enter the central square and ii) the duration of time spent there.

Ethical note

Mice were handled using handling tunnels to reduce stress which substantially improves the reliability of behavioural tests [1]. In the social competition assay (see below), males were divided by a mesh barrier to prevent direct contact. Furthermore, an observer remotely monitored each trial ready to intervene and stop the trial should escalated aggression occur. However, no intervention was necessary, since males mainly engaged in scent marking activity and tail rattling, which is a threat display.

Supplementary results

Communal litters were significantly larger than single litters in our experiment (CN = 11.1 ± 0.84; SN = 5.6 ± 0.5 [mean ± SE]; $t = 5.6$; $p < 0.001$). Taking advantage of the natural variation in litter sizes within each rearing background (SN = 1-7; CN = 7-14 pups) we looked for evidence of relationships between litter size and the main results *within* each rearing background (i.e. separately within the CN and SN treatment groups respectively).The social competition assay revealed that CN males scent marked a larger area when confronted with an unrelated opponent compared to a related opponent (see main text, Table 1d, Fig. 1). To check whether this result might be driven by litter size differences we first calculated the difference for each subject male between the area scent marked in front of an unrelated versus a related opponent. Then we plotted the relationship of this difference and litter size (Fig. S1-S2). The water barrier assay revealed that CN males had a shorter

latency to cross the water barrier than SN males (see main text, Table 2a). To check whether this result might have been influenced by litter size differences we plotted the relationship of the latency to cross the water barrier and litter size (Fig. S3-S4). When visually inspecting the figures, we are able to speculate that litter size *per se* might not be the main driver of the behavioural differences we report in the main text. Nevertheless, a targeted experiment is required to properly disentangle which factor of the early social environment experienced by communal and single reared males (including litter size variations) determines the observed behavioural differences.

Table S1: Overview of litters produced in the experiment, the number of dams in each round and the number of subjects used for each behavioural assay. In total we used eight breeding trios consisting of two females and one male. Each breeding trio was given the opportunity to breed three times but not all females bred successfully in each round. Thus, each female contributed a maximum of 2 litters to the same treatment group. Abbreviations: CN: communal nest; SN: single nest; SCA: social competition assay; WBA: water barrier assay; OFA: open field assay

Breeding trio	CN litters	SN litters	Nr. dams round 1	Nr. dams round 2	Nr. dams round 3	CN subjects in SCA	SN subjects in SCA	CN subjects in WBA and OFA	SN subjects in WBA and OFA
1	4	2	2	2	2	4	0	2	2
2	2	2	2	2	0	2	0	2	2
3	2	0	2	0	0	0	0	2	0
4	2	1	0	1	2	1	0	0	1
5	4	0	2	2	0	2	0	4	0
6	2	4	2	2	2	0	4	2	2
7	0	2	1	1	0	0	0	0	1
8	2	3	2	2	1	0	4	2	2
**Total**	**18**	**14**	**13**	**12**	**7**	**9**	**8**	**14**	**10**

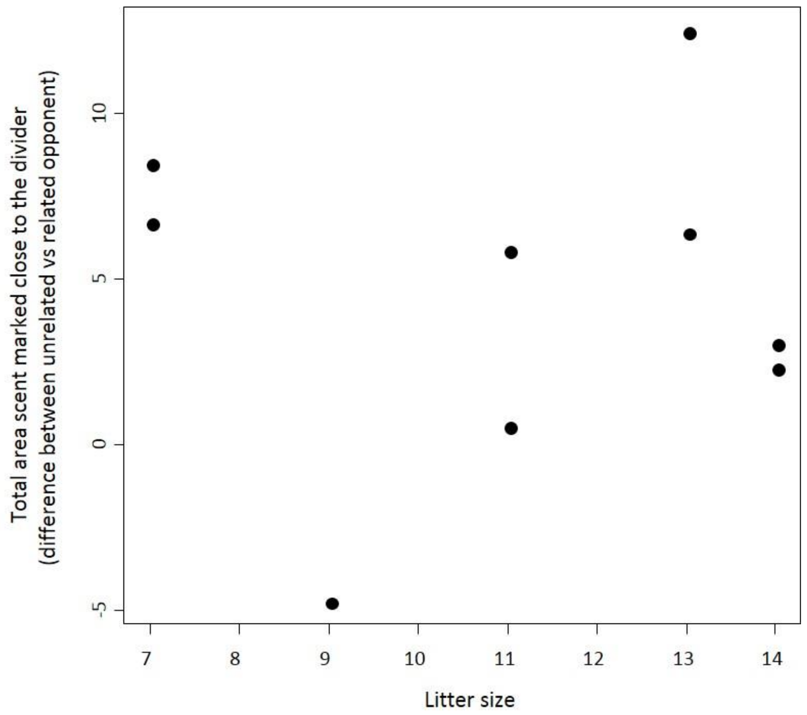

Fig. S1: Relationship of the difference between the area scent marked in front of an unrelated versus a related opponent and litter size for communal nest reared subjects.

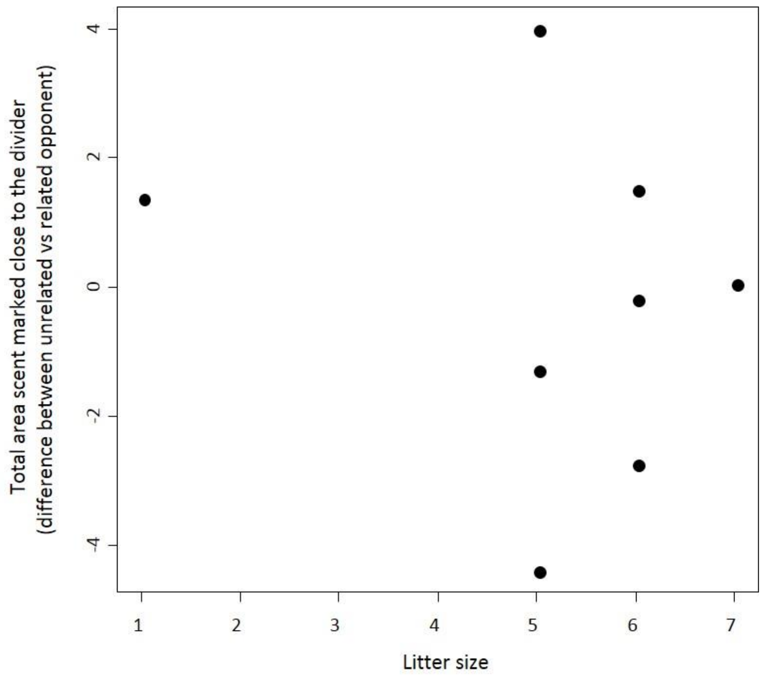

Fig. S2: Relationship of the difference between the area scent marked in front of an unrelated versus a related opponent and litter size for single nest reared subjects.

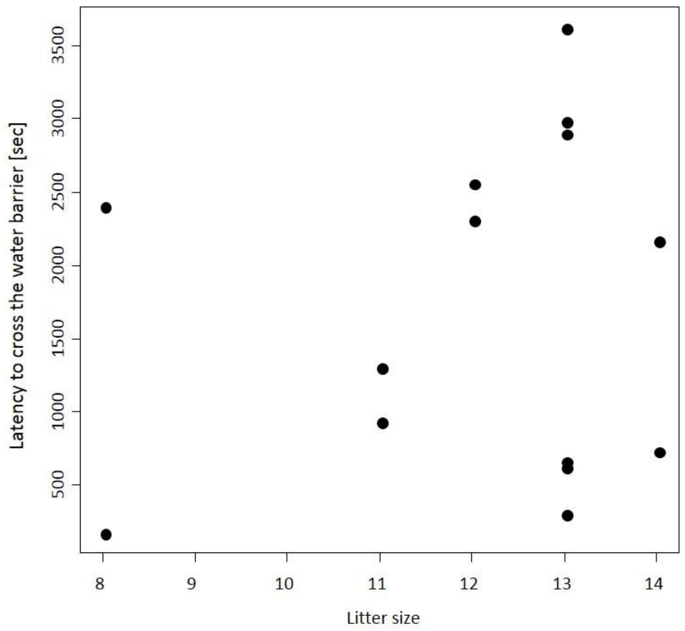

Fig. S3: Relationship of the latency to cross a water barrier and litter size for subjects reared in communal nests.

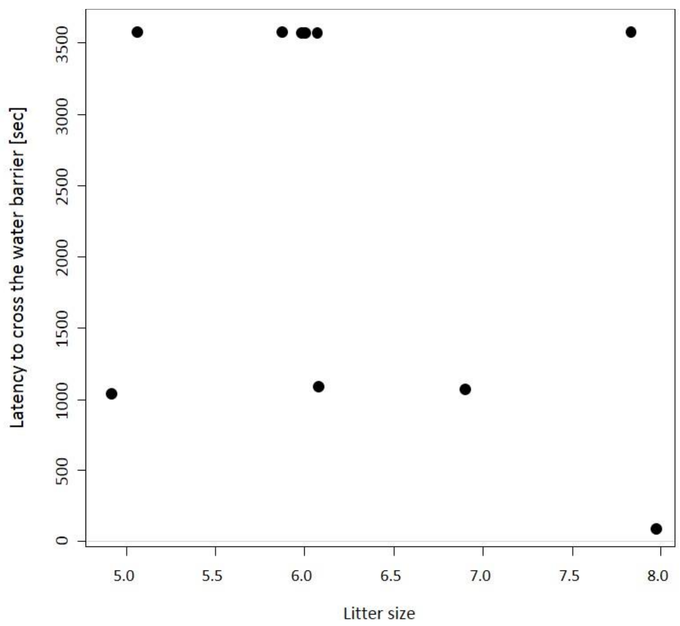

Fig. S4: Relationship of the latency to cross a water barrier and litter size for single nest reared subjects. To visualize overlapping values we added random noise to the y and x axis using the function jitter() in R.

Supplementary literature

1 Gouveia, K. & Hurst, J. L. Optimising reliability of mouse performance in behavioural testing: the major role of non-aversive handling. *Sci. Rep.* **7**, 12, doi:10.1038/srep44999 (2017).
